# The metabolic differences of anestrus, heat, pregnancy, pseudopregnancy, and lactation in 800 female dogs

**DOI:** 10.3389/fvets.2023.1105113

**Published:** 2023-02-02

**Authors:** Claudia Ottka, Katariina Vapalahti, Sebastian P. Arlt, Alexander Bartel, Hannes Lohi

**Affiliations:** ^1^PetBiomics Ltd., Helsinki, Finland; ^2^Department of Veterinary Biosciences, University of Helsinki, Helsinki, Finland; ^3^Department of Medical and Clinical Genetics, University of Helsinki, Helsinki, Finland; ^4^Folkhälsan Research Center, Helsinki, Finland; ^5^Clinic of Reproductive Medicine, Vetsuisse Faculty, University of Zurich, Zurich, Switzerland; ^6^Institute for Veterinary Epidemiology and Biostatistics, Faculty of Veterinary Medicine, Freie Universität Berlin, Berlin, Germany

**Keywords:** dog, metabolomics, metabolism, pregnancy, lactation, reproduction

## Abstract

**Introduction:**

Reproduction causes major hormonal and physiological changes to the female body. However, the metabolic changes occurring during canine reproduction are scarcely studied.

**Methods:**

In this cross-sectional study, we assessed the metabolic effects of canine reproductive status using a ^1^H NMR metabolomics platform optimized and validated for canine use. The study population consisted of a total of 837 healthy, intact female dogs in breeding age, of which 663 dogs were in anestrus, 78 in heat, 43 were pseudopregnant, 15 were pregnant, and 38 were lactating. The differences in metabolite profiles between these states were studied by the Kruskal-Wallis test with *post-hoc* tests performed using the Dunn's test, and visualized by box plots and a heatmap. The ability of the metabolite profile to differentiate pregnant dogs from non-pregnant ones was assessed by creating a multivariate Firth logistic regression model using forward stepwise selection.

**Results:**

Lactation, pregnancy and heat all were associated with distinct metabolic changes; pregnancy caused major changes in the concentrations of glycoprotein acetyls, albumin and creatinine, and smaller changes in several lipids, citrate, glutamine, and alanine. Pseudopregnancy, on the other hand, metabolically largely resembled anestrus. Lactation caused major changes in amino acid concentrations and smaller changes in several lipids, albumin, citrate, creatinine, and glycoprotein acetyls. Heat, referring to proestrus and estrus, affected cholesterol and LDL metabolism, and increased HDL particle size. Albumin and glycoprotein acetyls were the metabolites included in the final multivariate model for pregnancy detection, and could differentiate pregnant dogs from non-pregnant ones with excellent sensitivity and specificity.

**Discussion:**

These results increase our understanding of the metabolic consequences of canine reproduction, with the possibility of improving maternal health and ensuring reproductive success. The identified metabolites could be used for confirming canine pregnancy.

## 1. Introduction

The time around parturition is a challenging to the female body due to hormonal changes, whelping, nursing the puppies, milk production, and uterine involution (1). In this context, the transformation ability of the female dog's body is remarkable. It transforms from accommodating one individual to an incubation chamber for one or more additional individuals, and to an effective supplier of nutrients for newborn individuals *via* lactation. This process causes major alterations in the female body, and is governed by complex hormonal and neurological interactions. It is also a sensitive process, and multiple internal and external factors can cause severe harm to the pregnant female, its offspring, or both (2).

Since the mother's health is tightly linked with reproductive success, understanding the metabolic changes occurring during reproduction is essential for both the health of the mother and the health and normal development of the offspring. For example, the mother's nutritional needs for both micro- and macronutrients change during reproduction, and not meeting these needs can cause severe harm to the mother and its offspring (3, 4). Furthermore, differentiating physiological changes from pathophysiological ones are essential for monitoring the mother's health. For example, lower creatinine concentrations during pregnancy might mask renal dysfunction (5), and the physiologic elevation of several inflammatory parameters during pregnancy might lead to misinterpretation between pregnancy and inflammatory disease (6–12).

Metabolic profiling has been successfully used in humans to characterize specific changes occurring during pregnancy (12–17). However, the metabolic effects of the reproductive cycle, pregnancy and lactation have been scarcely studied in dogs, and metabolomics methods have not been utilized in studies in dog reproduction. Dog reproduction features differ from those of humans in several ways: dogs are monocyclic animals come in estrus only every 6–7 (5–12) months (18, 19). Furthermore, the corpora lutea persist for around 2 months after spontaneous ovulation in estrus, independent of whether a dog gets pregnant or not (19). The hormonal environment of non-pregnant dogs roughly mimics that of canine pregnancy and lactation (20, 21), and may cause similar symptoms to pregnancy and lactation in a state called pseudopregnancy (19, 22, 23). Implantation of the embryos occurs relatively late, around 18 days after the LH peak, i.e., during the late first trimester (6). Because of these differences, the metabolic similarities in canine and human reproduction need to be studied to evaluate whether findings from these species can be extrapolated to one another. While canine pregnancy and pseudopregnancy share similar hormonal environments and symptoms (20–23), little is known about the metabolic differences between these two states. Due to the similar symptoms of these conditions, diagnostic tests need to be used to confirm pregnancy (2). The usability of metabolic measures in pregnancy testing of dogs has not yet been evaluated.

Understanding the metabolic effects of the female reproductive cycle and the characteristics of metabolism in specific stages is crucial for monitoring health during these sensitive phases. Differentiating pathological changes from physiological adaptations will help detecting disorders early, and understanding the dog's metabolic needs can help in providing optimal nutrition and thereby ensuring reproductive success. This study aims to characterize differences in metabolism of healthy unspayed bitches in anestrus, heat, pseudopregnancy, pregnancy, and lactation. Additionally, we aim to assess whether metabolite data can be used to differentiate pregnant dogs from non-pregnant ones.

## 2. Materials and methods

The study samples and data were derived from a previously collected sample set of 6,306 serum/plasma samples from pet dogs. Details of this sample set, their collection and NMR analysis are described elsewhere (24). Briefly, the samples were collected by cephalic venipuncture from privately owned dogs across Finland in 2017–2018. The samples were immediately cooled after separation from blood cells and stored at −80°C until proton nuclear magnetic resonance (^1^H NMR) spectroscopy analysis. The owners of the dogs completed questionnaires that covered the dog's signalment, current health, diet, exercise, stress, and reproductive cycle phase. Participation was voluntary, and the dog owners gave signed informed consent. Blood sampling followed the national and European Union's legislation and was approved by the Animal Ethics Committee of State Provincial Office of Southern Finland, permit number: ESAVI/7482/04.10.07/2015, approval date: 9.10.2015.

### 2.1. ^1^H NMR metabolomics

**Figure 1:**
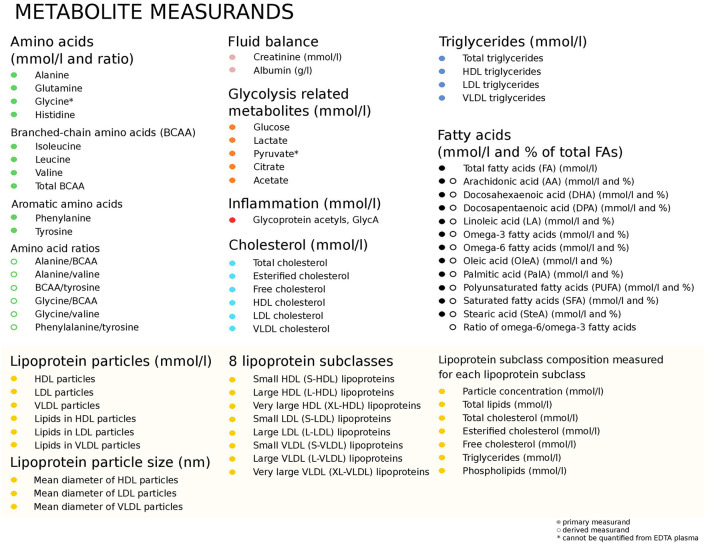
Measurands quantified using the validated (24) canine NMR metabolomics platform. BCAA, Branched-chain amino acids; HDL, High-density lipoprotein; LDL, Low-density lipoprotein; VLDL, Very low-density lipoprotein.

The samples were analyzed using a validated, dog-specific ^1^H NMR metabolomics platform quantifying 123 measurands in absolute units (24). The quantified measurands are presented in Figure 1. Details about the method and its validation is provided elsewhere (24–27).

Briefly, the method utilizes a Bruker AVANCE III HD 500 NMR spectrometer equipped with a 5 mm triple-channel (^1^H, ^13^C, ^15^N) z-gradient Prodigy probe head and a cooled high-throughput sample changer SampleJet (Bruker Corp., Billerica, Massachusetts, USA). Sample preparation begins with light mixing of the sample and centrifugation to remove possible precipitate (25). The highly automated process then continues by transfer of each sample into individual NMR tubes and mixing with sodium phosphate buffer using a PerkinElmer JANUS Automated Workstation equipped with an 8-tip dispense arm with Varispan (PerkinElmer Inc., Waltham, Massachusetts, USA) (26). The NMR spectra are acquired automatically from each sample using standardized parameters and are automatically processed using in-house scripts optimized for dogs (24–27). Result generation is based on regression modeling and includes integrated quality control (27).

### 2.2. Sample inclusion

**Figure 2:**
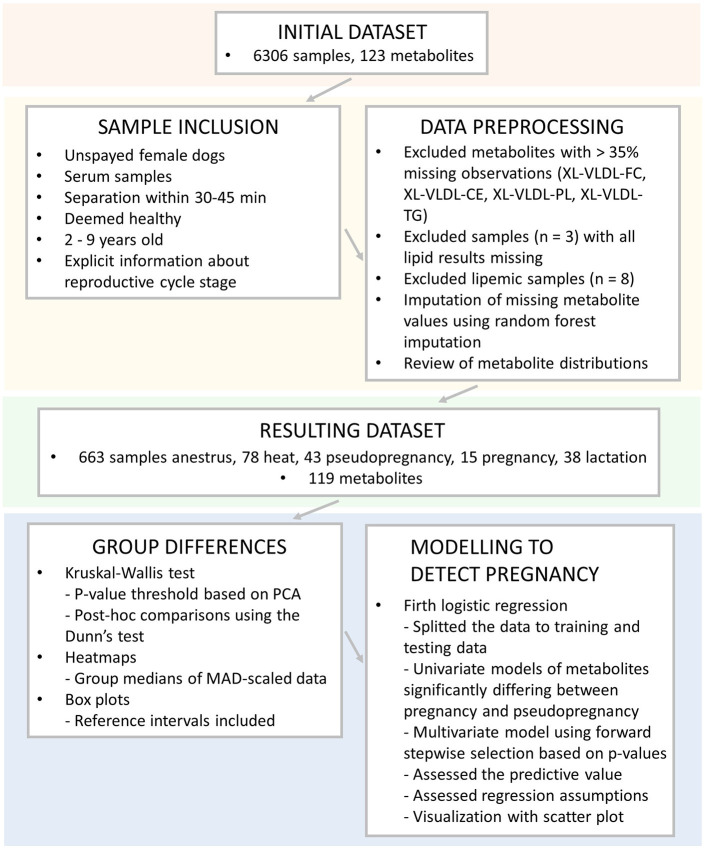
Overview of the used samples, inclusion criteria, data preprocessing, and statistical analyses utilized in this study. XL-VLDL, Very large very low-density lipoprotein; -FC, free cholesterol; -CE, esterified cholesterol; -PL, phospholipids, -TG, triglycerides; PCA, Principal component analysis; MAD, median absolute deviation.

The overview of the used samples, inclusion criteria, data preprocessing and statistical analyses are presented in Figure 2.

A review of sample inclusion for statistical analyses was conducted in Microsoft Office Excel (Microsoft Corp., Redmond, WA, US). Only samples from unspayed female dogs were used from the previously collected dataset (24). We only included serum samples to diminish the effects of different sample types on the results. Samples for which serum separation from blood cells had not been conducted according to sample tube manufacturer's recommendations (separation of serum within 30–45 mins of blood sampling) were removed from the dataset.

The dogs' owner-reported health status was carefully reviewed, and only dogs deemed healthy were included in the study. For example, dogs with minor eye diseases, such as distichiasis or mild or moderate joint dysplasia observed in routine breeding-related x-rays without symptoms or treatment, were considered healthy. To evaluate only data of dogs in typical breeding age, we included samples from animals aged from over 2 to under 9 years old. The owner-reported information about the reproductive cycle stage status (in heat, pregnant, lactating or pseudopregnant) at the time of sample collection was carefully reviewed. Only samples from dogs with specific cycle stage information were included. Samples from dogs with unclear cycle stages or unknown reproductive status were excluded, such as dogs mated without pregnancy diagnosis. Dogs not reported to be in heat, pregnant, lactating or pseudopregnant were included in the anestrus group.

Finally, the data were checked for differences in age and fasting duration in R Project for Statistical Computing (RRID:SCR_001905) (28) using the Kruskal-Wallis test using the package “stats,” version 3.6.2 (28). Medians and median absolute deviations (MAD) were calculated using the package “stats,” version 3.6.2 (28), with the help of the package “dplyr,” version 1.0.7 (29). No significant differences in age or fasting duration were observed in the anestrus, heat, pseudopregnancy, pregnancy, and lactation -groups. The final dataset included a total of 837 samples. Of these samples, 663 were from dogs in anestrus, 78 from dogs in heat, 43 from pseudopregnant, 15 from pregnant, and 38 from lactating dogs.

### 2.3. Data preprocessing and statistical methods

Data preprocessing and statistical analyses were conducted in R 4.1.2 (28). Data preprocessing was started by reviewing missing observations. Metabolite measurands with over 35% missing observations were removed. These measurands included the following very large very low-density lipoprotein measurands (XL-VLDL): XL-VLDL free cholesterol, XL-VLDL esterified cholesterol, XL-VLDL phospholipids, and XL-VLDL triglycerides. Three dogs that missed all lipid results and were removed from the study. The data were reviewed for outliers, and eight lipemic samples with total cholesterol over 15 mmol/l or total triglycerides over 1.5 mmol/l were removed.

Most of the remaining metabolite measurands had under 1% (range: 0–27%) missing observations. The remaining missing metabolite values were imputed using random forest (RF) imputation using the package “missForest” version 1.4 (30). Default values of the missForest function were used with the maximum number of iterations set to ten and the number of trees set to 100. The normalized root mean squared error (NRMSE) value was used to check the performance of RF imputation. The true imputation error was 3.39e−13, which indicates excellent performance. The distributions of all metabolite measurands were checked visually individually for each measurand. Most of the measurands were not normally distributed.

The significance threshold for hypothesis testing was obtained from Bonferroni correction based on the number of principal components explaining over 95% of the variation in the metabolite data (27). This method was used since many of the metabolite results are intercorrelated, and Bonferroni correction should be conducted based on individual comparisons. Principal component analysis was conducted on the dataset using the package “stats,” version 3.6.2 (28). Nineteen principal components explained 95% of the variation in the data, resulting in a significance threshold of p < 0.0026 (0.05/19).

Differences in metabolite measurands between the five reproductive state groups were analyzed using the Kruskal-Wallis test using the package “stats,” version 3.6.2 (28). The abovementioned significance threshold of *p* < 0.0026 was used in these analyses. *Post-hoc* comparisons were conducted using the Dunn test using the package “FSA” (version 0.8.20) (31). *p*-Values were adjusted using Bonferroni correction based on the number of individual comparisons. The *p*-value threshold was set at *p* < 0.05.

Data visualization included box plots and a heatmap. Box plots were created to visualize the distributions in each group, and conducted using the packages “ggplot2” (32) and “ggpubr” (version 0.4.0) (33). To understand the magnitude of the differences, the serum all dogs' reference intervals of the NMR method (24) were incorporated into the box plots. A heatmap was created to visualize the overall differences in metabolite values in the studied groups. First, the metabolite data was MAD-scaled. Medians of the MAD-scaled data were calculated for all groups, and metabolite measurands significant in the Kruskal-Wallis test were incorporated in the heatmap. The packages “ComplexHeatmap” (34), “grid” (28), “dplyr”, version 1.0.7 (29), “BiocManager,” version 1.30.16 (35), and “RColorBrewer” version 1.1-2 (36) were utilized in the creation of the heatmap. The heatmap and box plots were finalized in Inkscape (37).

To study whether metabolites could differentiate pregnant dogs from non-pregnant ones, we created Firth logistic regression (38) models using the package “logistf,” version 1.24.1 (39). Firth logistic regression was used due to the small and imbalanced groups. Pregnancy status served as the dependent variable, and the metabolite was the independent variable. To create these models, the data from pregnant and pseudopregnant dogs were split into training and testing data using the packages “caret,” version 6.0-90 (40) and “tidyverse” (41). The training data covered 80%, and the testing data 20% of the observations. The model building was done using the training data.

First, we created univariate Firth logistic regression models for each measurand showing significant differences in metabolite values between pregnancy and pseudopregnancy in the Kruskal-Wallis test. The univariate models are shown in Supplementary Data S5.

Then, we created a multivariate Firth logistic regression model with forward stepwise selection by adding sequentially the measurands that received the lowest *p*-value when added to the model, until no additional metabolite measurands reached a *p*-value threshold of <0.05. Only the metabolites showing significant differences between pregnancy and pseudopregnancy in the Kruskal-Wallis test were utilized in model building. Before modeling, we tested whether the metabolites had any significant interactions to be included in the model building. The multivariate model and the forward stepwise selection process are shown in Supplementary Data S6.

We evaluated carefully, whether the model met the logistic regression assumptions and whether the model was appropriately fitted. Multicollinearity of the model was checked using variance inflation factor (VIF) with a cutoff of 10, using the package “regclass,” version 1.6 (42). The linearity of the logit was checked by adding the interaction term of the independent variable and its natural logarithm to the model. If the interaction term significantly contributed to the model (*p* < 0.05), linearity of the logit was considered not to be present. The presence of influential observations was evaluated by calculating the residuals with the help of the package “cmvnorm,” version 1.0-7 (43).

The testing data was used to evaluate, how well the model can differentiate pregnant dogs from pseudopregnant ones in samples not included in model creation. This was conducted using the package “pROC” (44). A confusion matrix was generated to observe, which amount of pregnant and pseudopregnant dogs were correctly classified by the model, and model sensitivity and specificity were evaluated. Since the testing dataset was small, we wanted to assess further, how well the model differentiates pregnant dogs from non-pregnant ones in a larger number of female dogs. For this purpose, we created the AE dataset with the samples of dogs in anestrus (*n* = 663) and all 15 pregnant dogs in the data, including both the pregnant dogs in the training and testing data. Similarly to the testing data, a confusion matrix was generated, and the sensitivity and specificity of the model evaluated in the AE data. A plot combining the receiver operating characteristic (ROC) curves and their area under the curve (AUC) of the testing, training and AE data was generated and evaluated. The plot was finalized in Inkscape (37).

To visualize how the final multivariate model functioned and to visualize how pregnant, pseudopregnant and anestrus samples cluster using the metabolites included in the final multivariate model, we created a scatter plot using the package “tidyverse” (41). The scatter plot was finalized in Inkscape (37).

## 3. Results

### 3.1. Group characteristics

Characteristics of the study groups are presented in Table 1. No significant differences in ages or fasting duration between the reproductive stage groups were observed. All groups consisted of a large variety of different breeds.

**Table 1:** Characteristics of the study groups.

**Group**	***N***	**Age years**		**Fasting hours**		***N* breeds**
		**Median (MAD)**	***p***	**Median**	***p***	
Anestrus	663	4.1 (1.9)		>12		189
Heat	78	4.2 (2.1)		>12		52
Pseudopregnancy	43	4.2 (2.2)	0.73	>12	0.65	30
Pregnancy	15	3.5 (1.7)		>12		13
Lactation	38	3.9 (1.6)		>12		30

P-values of group differences are derived from the Kruskal-Wallis test. MAD, median absolute deviation.

### 3.2. Heat, pregnancy and lactation are associated with distinct metabolic characteristics

**Figure 3:**
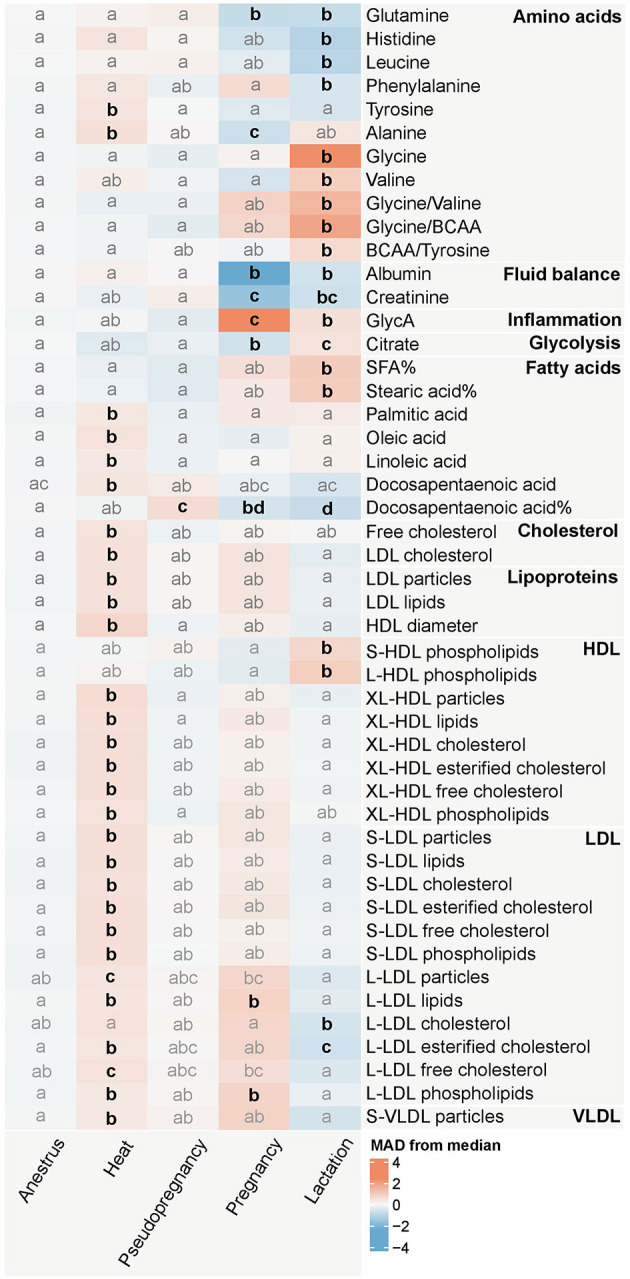
Heatmap of the measurands significantly (*p* < 0.0026) associated with the reproductive state in the Kruskal-Wallis test. The colors represent the median of each reproductive state group in median absolute deviation (MAD) scaled data. The intensity of the color increases proportionally with the magnitude each reproductive state differs from the overall median metabolite value. The change in magnitude is expressed as the amount of MAD:s the median of each reproductive state group differs from the overall median of the metabolite. Blue hues indicate that the median MAD of the group is lower, and red hues indicate that the median MAD of the group is higher than the overall median of the dataset. The letters indicate similarity of groups: groups with the same letters do not differ significantly from each other, whereas groups with different letters differ significantly (*p* < 0.05) from each other for the metabolite in question in the Dunn's test with Bonferroni correction, which was performed as a *post-hoc* test for the Kruskal-Wallis test. Emboldened, black letters indicate significant differences from anestrus. Anestrus *n* = 663, heat *n* = 78, pseudopregnancy *n* = 43, pregnancy *n* = 15, lactation *n* = 38. BCAA, Branched-chain amino acids; GlycA, Glycoprotein acetyls; SFA, Saturated fatty acids. %, percentage of the molar concentration of total fatty acids. Fatty acids without the symbol % are derived from the absolute (mmol/l) concentration of the fatty acid in question. LDL, Low-density lipoprotein; HDL, High-density lipoprotein; VLDL, Very low-density lipoprotein; S, small; L, large; XL, very large.

The concentrations of multiple lipids, such as the molar concentrations of the fatty acids palmitic acid (PalA), oleic acid (OleA), linoleic acid (LA), and docosapentaenoic acid (DPA), as well as free cholesterol, low-density lipoprotein (LDL) cholesterol, LDL particles and lipids as well as high-density lipoprotein (HDL) particle size were increased during heat compared to anestrus (Figure 3; Supplementary Data S2, S3). In a more detailed analysis of lipoprotein subclasses, XL-HDL particles, all LDL particle subtypes and their components, excluding triglycerides, were increased. Additionally, the particle concentration of small very low-density lipoprotein (S-VLDL) particles, and the concentrations of the amino acids tyrosine and alanine were increased.

Minimal differences compared to anestrus were observed during pseudopregnancy, with only the relative concentration of DPA being increased (Figure 3; Supplementary Data S2, S3). The concentrations of the lipids and amino acids changed in heat were near the concentrations observed in anestrus.

Conversely, pregnancy caused changes with some of the largest magnitudes, with the concentrations of albumin and GlycA differing from the median of the dataset with over 2 MAD:s (Figure 3; Supplementary Data S2, S3). Additionally, the concentrations of the amino acids glutamine and alanine, creatinine, citrate, and the relative DPA concentration, decreased during pregnancy compared to anestrus. The lipid and phospholipid concentrations of large LDL (L-LDL) particles remained elevated after heat during pregnancy.

Lactation was associated with changes in multiple measurands in multiple metabolite groups (Figure 3; Supplementary Data S2, S3). Differences from anestrus included decreased concentrations of the amino acids glutamine, histidine, leucine, and phenylalanine, albumin, creatinine, the relative concentration of DPA, and the concentration of esterified cholesterol in L-LDL particles. Conversely, the concentrations of the amino acids glycine and valine, as well as the amino acid ratios glycine/valine and glycine/branched-chain amino acids (BCAA), GlycA, citrate, the relative concentration of saturated fatty acids, and stearic acid (SteA), as well as phospholipids in S-LDL and L-LDL particles, were increased compared to anestrus.

### 3.3. Metabolite concentrations rarely fall outside reference intervals

**Figure 4:**
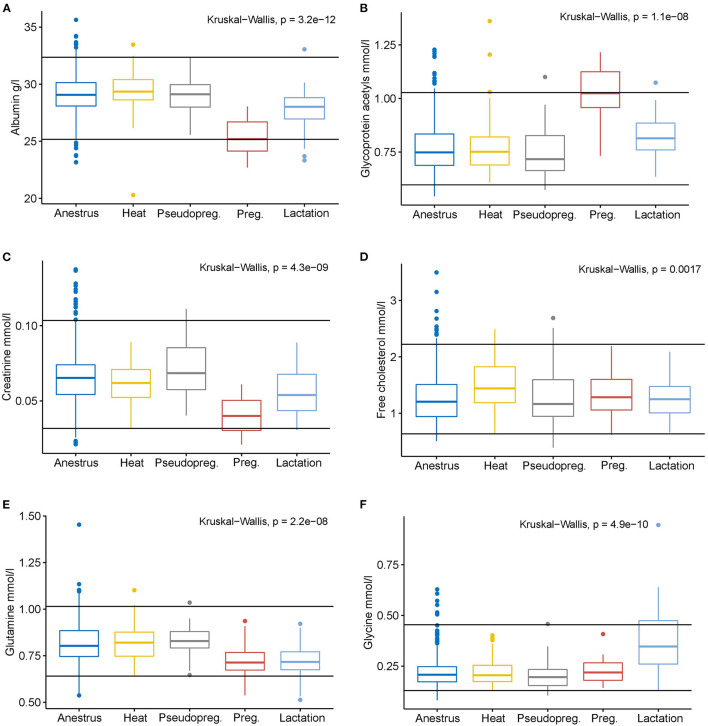
Box plots of selected measurands with significantly different concentrations from anestrus in one or more of the other reproductive cycle phases according to the Kruskal-Wallis test. The black horizontal lines indicate the serum all dogs' reference intervals of the NMR method (Ottka et al. 2021). Anestrus *n* = 663, heat *n* = 78, pseudopregnancy (Pseudopreg.) *n* = 43, pregnancy (Preg.) *n* = 15, lactation *n* = 38. **(A)** Albumin, **(B)** Glycoprotein acetyls, **(C)** Creatinine, **(D)** Free cholesterol, **(E)** Glutamine, **(F)** Glycine.

While different phases of the reproductive cycle showed distinct metabolic differences, the metabolite concentrations in each group rarely exceeded or undercut the all dogs' serum reference intervals of the NMR method (Figure 4; Supplementary Data S3). Only the median of pregnant dogs for albumin and GlycA reached the method's lower and upper reference limits, respectively. Other changes were more subtle in magnitude, especially compared to the reference intervals of the method. However, it must be noted, that 152 (18.2%) of the dogs included in this study were used in the generation of the all dogs' reference intervals (24).

### 3.4. GlycA and albumin can differentiate pregnant dogs from non-pregnant ones

In the univariate Firth logistic regression models, the measurands with strongest contribution to predicting the inclusion to the pseudopregnant and pregnant groups were albumin (*p* < 0.001), creatinine (*p* < 0.001), GlycA (*p* < 0.001), glutamine (*p* < 0.001), and alanine (*p* = 0.008). The relative concentration of DPA (*p* = 0.734) was not a suitable predictor of group inclusion in these univariate models (Supplementary Data S5).

**Figure 5:**
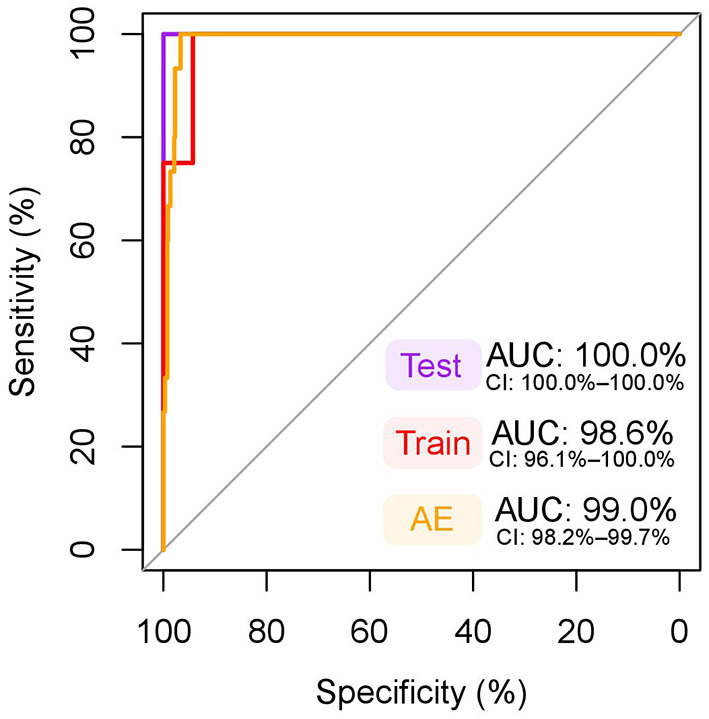
ROC curve of the multivariate model for classification of pregnant dogs. The dependent variables included in this model were albumin and glycoprotein acetyls (GlycA). An area under the curve (AUC) nearing 100% is considered excellent. Test: receiver operating characteristic (ROC) curve, AUC, and its 95% confidence intervals (CI) in testing data (pseudopregnant *n* = 8, pregnant n = 3), used to test the model fit in a sample set independent from the training data. Note, that the CI for an AUC of 100% is not defined. Train: ROC curve, AUC, and its 95% CI in training data (pseudopregnant *n* = 35, pregnant *n* = 12), which was used to create the model. The testing and training datasets were randomly split prior to model generation from data including all pregnant (*n* = 15) and pseudopregnant (*n* = 43) dogs to cover 20% and 80% of the samples, respectively. AE: ROC curve, AUC, and its 95% CI in the data including dogs in anestrus (*n* = 663) and all pregnant (*n* = 15) dogs. This data was used to test the model in a larger dataset of female dogs.

The final multivariate model created by forward stepwise selection included albumin and GlycA as predictors (Supplementary Data S7). None of the interaction terms reached statistical significance (*p* < 0.05), and age and fasting duration had no significant effect on the model, and therefore these terms were not included in the final model. The model met the assumption of linearity of the logit and multicollinearity was not observed. The model's AUC on the training data was 98.6% (Figure 5), which is considered excellent. The model performed exquisitely on the testing data with all pseudopregnant (*n* = 8) and pregnant (*n* = 3) dogs classified correctly (Supplementary Data S8), making the sensitivity, specificity, and AUC on the testing data 100% (Figure 5). The model performed well also in classifying pregnant (*n* = 15) dogs and dogs in anestrus (*n* = 663), having an AUC of 99.0% (Figure 5), a sensitivity of 93.3% and a specificity of 97.3% (Supplementary Data S9).

**Figure 6:**
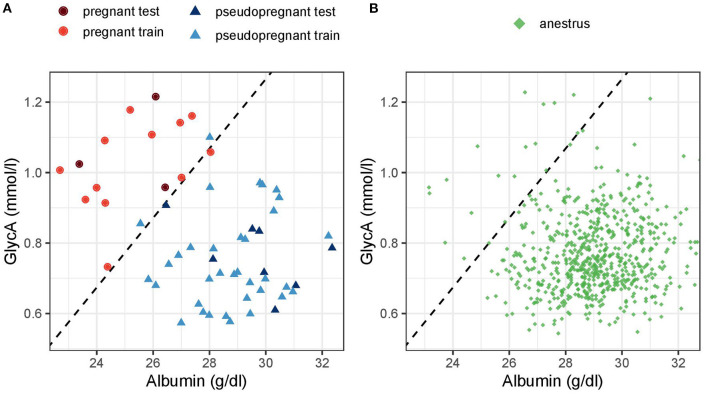
Scatter plot of **(A)** pregnant and pseudopregnant dogs in the testing and training data and **(B)** dogs in anestrus based on albumin and glycoprotein acetyls (GlycA) concentration. The dashed line represents the decision threshold for a cutoff of 0.5 of the multivariate Firth logistic regression model. Dogs above the dashed line (the top left) would be classified as pregnant and dogs below it (down right) as non-pregnant.

Figure 6 highlights the distinct clustering of the samples of pregnant dogs against pseudopregnant dogs and dogs in anestrus based on serum albumin and GlycA concentrations and visualizes how the multivariate model functions.

## 4. Discussion

This study identified several metabolic differences in healthy, unspayed female dogs during anestrus, heat, pseudopregnancy, pregnancy and lactation, many of which mimic those observed in humans. Uncovering these changes increases our understanding of the metabolic consequences of female reproduction and is crucial for accurately interpreting laboratory values in female dogs. Furthermore, we developed a multivariate Firth logistic regression model based on these metabolites that performed excellently in detecting pregnant dogs. This finding suggests, that metabolite concentrations could be used as an additional indirect method of pregnancy testing in healthy dogs with a high sensitivity and specificity.

Heat, referring to proestrus and estrus, was characterized by several changes in lipid concentrations, such as increases in LDL measures, XL-HDL particle measures, S-VLDL particles, free cholesterol, the saturated fatty acid PalA, the monounsaturated fatty acid OleA, and the polyunsaturated fatty acids LA and DPA. Previous studies on changes in lipid concentrations during the estrous cycle have had conflicting results, with some studies reporting no changes and some observing several lipid changes during the follicular phase (16, 17, 45–49). These results can be partly explained by methodological differences, such as measuring different lipid parameters. Furthermore, the magnitude of the lipid changes we observed was relatively small, and all changes remained well within the reference intervals, and thus observing these changes requires large sample sizes. In this study, the concentrations of the amino acids tyrosine and alanine were also increased during heat. Similar changes in amino acids have been previously reported in humans during the follicular phase, the follicular phase being characterized by higher amino acid levels than the luteal phase (16, 17, 50). These changes have been suggested to occur due to changes in anabolic and catabolic activity during the menstrual cycle (16, 17). Furthermore, feeding tyrosine during heat has been recommended, and while there are no documented benefits of its use (51), the possible increased ingestion of this amino acid might affect its plasma concentration.

Pregnancy greatly influenced the concentrations of the inflammatory markers GlycA and albumin. Pregnancy is known to cause a major burden on the female immune system, which has to provide an adequate immune response to protect both the female and its offspring, and tolerate inflammatory reactions caused by implantation, endometrial invasion, placental development, and growth of the fetus (2, 6, 11). Different inflammatory markers have been shown to peak at various stages of canine pregnancy, but all changes seem to occur after implantation (6). Since inflammatory markers are routinely used to detect inflammation, the effects of pregnancy should always be considered in result interpretation, and pregnancy-specific reference intervals should be determined to aid in this interpretation challenge (10).

The decrease in creatinine concentration during pregnancy due to increased glomerular filtration rate is a well-known phenomenon and has important practical implications (2, 5). Since glomerular filtration rates based on blood creatinine measurements are commonly used when diagnosing renal disease (52, 53), the physiological change in its concentration during pregnancy can mask renal insult and lead to possibly severe renal diseases from being underdiagnosed during pregnancy (5). Thus, the reproductive state needs to be considered when assessing blood creatinine concentrations. The slightly lower serum citrate levels observed in pregnant dogs are likely caused by the higher glomerular filtration rate during pregnancy, causing increased urinary citrate excretion (54–56). However, previous studies have not identified decreased plasma citrate concentrations during pregnancy (12).

The concentrations of the amino acids glutamine and alanine were also decreased during pregnancy. Changes in amino acid concentrations during pregnancy have previously been observed in several species, with a general trend of amino acid levels decreasing during pregnancy (12, 57–63). The recommendations for protein intake are higher during canine pregnancy (4), and the decreased amino acid levels are thought to arise from increased postprandial insulin (61), and high placental amino acid uptake (63).

Certain polyunsaturated fatty acids (PUFA), such as docosahexaenoic acid (DHA), arachidonic acid (AA), and eicosapentaenoic acid (EPA), are known to be required for optimal development of the neonate, especially regarding the neural system (3, 4, 64–66). In this study, the molar concentrations of all studied PUFA, including DHA, DPA, AA, and LA in serum of pregnant and lactating bitches were similar to anestrus, and only the relative concentration of DPA was lower during pregnancy and lactation than during anestrus. This finding suggests that PUFA concentrations are relatively well preserved in canine plasma during pregnancy and lactation. Further studies are needed to evaluate, whether bitches with low serum DPA or their offspring would benefit from PUFA supplementation.

In lactating dogs, the concentrations of GlycA, albumin and creatinine were nearing the same values as during anestrus after the striking changes during pregnancy and remained only slightly different from anestrus and well within the established reference intervals. It needs to be taken into account that after parturition also uterine involution and repair of the former placental sites in the uterus occur (67). These phenomena may result in elevated inflammatory markers for a certain time, even in dogs with a normal puerperium. In late lactation, GlycA or albumin concentration falling outside reference intervals in lactating bitches may not be physiological and should be evaluated further. Lactating dogs also had several differences in serum amino acid concentrations compared to dogs in anestrus: glutamine, histidine, leucine and phenylalanine were decreased, whereas glycine and valine, as well as the ratios of glycine to valine and glycine to total BCAA, were increased. These changes likely reflect the high amino acid utilization to meet the high protein concentrations of secreted milk (68–70). Sufficient protein intake is essential in lactating bitches to meet the high protein demands of lactation (4). Further studies are needed to evaluate whether serum amino acid concentrations might reflect the protein requirements of lactation. However, in this study, the amino acid concentrations of lactating bitches rarely fell outside the established reference intervals.

Furthermore, lactation caused multiple changes in energy metabolism, and most of the changes in lipid concentrations during pregnancy were reversed during lactation. Changes in lipid and glucose metabolism are known to occur in breastfeeding human mothers, and breastfeeding mothers generally have lower lipid levels than non-breastfeeding mothers (71). Energy metabolism during lactation is characterized by a higher metabolic rate, higher energy expenditure and mobilization of fat stores to meet the lipid needs of milk production, while carbohydrates are still preferred as an energy source (3, 4, 72–76). Milk is rich in lipids (68–70), and saturated fatty acids dominate its fatty acid composition (68). The increased production and mobilization of saturated fatty acids might cause the higher relative concentrations of saturated fatty acids in the serum of lactating dogs observed in this study.

Except for albumin and GlycA during pregnancy, the changes in metabolite concentrations in any studied reproductive states did not fall outside the all dogs' reference intervals. Thus, established reference intervals for most metabolites seem to be valid for female dogs in all phases of reproduction. However, special attention should be given to interpreting GlycA, albumin, and creatinine results.

Interestingly, pseudopregnancy was associated with hardly any metabolic changes compared to anestrus. This finding suggests that the hormonal changes alone do not cause massive metabolic changes but metabolic changes during pregnancy and lactation are specifically caused by implantation, fetal growth and nursing of puppies. However, since the exact cycle days of the dogs were unknown, the pseudopregnancy group probably includes both dogs in late metestrus and early anestrus, possibly having different hormonal backgrounds.

Indeed, the greatest limitation of this study was that both the health of the dog and the reproductive state was owner-reported. While the owner-reported health information was evaluated by a veterinary professional, incorrect reporting of the disease status of the dogs could have occurred. Furthermore, misclassifications of reproductive state are possible. To minimize potential bias, unclear cases based on owner-reported status were removed from the dataset. Nevertheless, no clinical or laboratory examinations for cycle staging were performed. Since no information about heat-related symptoms or cycle day were reported, estrus and proestrus cannot be differentiated, and potential dependences such as the relation to the day of ovulation or interactions with rising progesterone concentrations cannot be assessed in this study. Since no specific definition of the term pseudopregnancy was provided for the owners, it is unclear whether owners classified their dogs as pseudopregnant based on timing or overt signs of pseudopregnancy. Therefore, the dogs reported to be pseudopregnant may include dogs in late metestrus and early anestrus, and dogs reported to be in anestrus might include dogs in metestrus. Furthermore, pregnancy and lactation duration was mostly unknown, and parity and litter size was unknown. Hence, it is not evident at which time point during pregnancy and lactation the observed changes occur and whether parity or litter size affects them. Some changes may even be masked, for example if parameters change significantly only in a specific phase of pregnancy or lactation. Thus, further studies with definitively determined oestrus cycle phase and detailed information on the litter may reveal the effects of female reproduction on metabolism in greater detail. Nevertheless, the changes observed in each group align with previous literature on other species.

The created multivariate Firth logistic regression model was able to differentiate healthy pregnant dogs from non-pregnant dogs. While its sensitivity and specificity were good in this dataset, the model has limitations. Similarly as to other acute-phase protein -based pregnancy tests, they are only usable for pregnancy determination in definitively healthy individuals (2, 6, 11). Inflammatory conditions, such as pyometra, which is a severe differential diagnosis for pregnancy and pseudopregnancy, affect acute-phase proteins and severe misdiagnoses might happen if the bitches' health status is not confirmed (2, 6, 11). Similarly, other acute-phase protein-based pregnancy tests are only usable for pregnancy determination in definitively healthy individuals (2, 6, 11). Furthermore, whether the model performs as well in pregnant and non-pregnant dogs' samples taken on similar cycle days should be further studied. Finally, it should be studied during which time period in pregnancy GlycA increases and when albumin decreases.

This study identified several metabolic changes in healthy bitches during anestrus, heat, pseudopregnancy, pregnancy and lactation. Understanding these changes is crucial for accurately interpreting laboratory values and increases our understanding on the metabolic consequences of female reproduction. A multivariate Firth logistic regression model based on the inflammatory markers albumin and glycoprotein acetyls was able to differentiate pregnant dogs from non-pregnant ones. This finding suggests, that after further study, a model based on metabolite data could be used as a method of pregnancy testing in healthy dogs.

## Data availability statement

The raw data supporting the conclusions of this article will be made available by the authors, without undue reservation.

## Ethics statement

The animal study was reviewed and approved by Animal Ethics Committee of State Provincial Office of Southern Finland. Written informed consent was obtained from the owners for the participation of their animals in this study.

## Author contributions

CO: conceptualization, data curation, formal analysis, investigation, methodology, visualization, writing—original draft, and writing—review and editing. KV and SA: methodology and writing—review and editing. AB: methodology, visualization, and writing—review and editing. HL: conceptualization, funding acquisition, project administration, resources, supervision, and writing—review and editing. All authors contributed to the article and approved the submitted version.
